# Mitigating Cognitive Biases in Clinical Decision-Making Through Multi-Agent Conversations Using Large Language Models: Simulation Study

**DOI:** 10.2196/59439

**Published:** 2024-11-19

**Authors:** Yuhe Ke, Rui Yang, Sui An Lie, Taylor Xin Yi Lim, Yilin Ning, Irene Li, Hairil Rizal Abdullah, Daniel Shu Wei Ting, Nan Liu

**Affiliations:** 1 Centre for Quantitative Medicine Duke-NUS Medical School Singapore Singapore; 2 Department of Anesthesiology Singapore General Hospital Singapore Singapore; 3 Information Technology Center University of Tokyo Tokyo Japan; 4 Singapore Eye Research Institute Singapore National Eye Centre Singapore Singapore; 5 Institute of Data Science National University of Singapore Singapore Singapore

**Keywords:** clinical decision-making, cognitive bias, generative artificial intelligence, large language model, multi-agent

## Abstract

**Background:**

Cognitive biases in clinical decision-making significantly contribute to errors in diagnosis and suboptimal patient outcomes. Addressing these biases presents a formidable challenge in the medical field.

**Objective:**

This study aimed to explore the role of large language models (LLMs) in mitigating these biases through the use of the multi-agent framework. We simulate the clinical decision-making processes through multi-agent conversation and evaluate its efficacy in improving diagnostic accuracy compared with humans.

**Methods:**

A total of 16 published and unpublished case reports where cognitive biases have resulted in misdiagnoses were identified from the literature. In the multi-agent framework, we leveraged GPT-4 (OpenAI) to facilitate interactions among different simulated agents to replicate clinical team dynamics. Each agent was assigned a distinct role: (1) making the final diagnosis after considering the discussions, (2) acting as a devil’s advocate to correct confirmation and anchoring biases, (3) serving as a field expert in the required medical subspecialty, (4) facilitating discussions to mitigate premature closure bias, and (5) recording and summarizing findings. We tested varying combinations of these agents within the framework to determine which configuration yielded the highest rate of correct final diagnoses. Each scenario was repeated 5 times for consistency. The accuracy of the initial diagnoses and the final differential diagnoses were evaluated, and comparisons with human-generated answers were made using the Fisher exact test.

**Results:**

A total of 240 responses were evaluated (3 different multi-agent frameworks). The initial diagnosis had an accuracy of 0% (0/80). However, following multi-agent discussions, the accuracy for the top 2 differential diagnoses increased to 76% (61/80) for the best-performing multi-agent framework (Framework 4-C). This was significantly higher compared with the accuracy achieved by human evaluators (odds ratio 3.49; *P*=.002).

**Conclusions:**

The multi-agent framework demonstrated an ability to re-evaluate and correct misconceptions, even in scenarios with misleading initial investigations. In addition, the LLM-driven, multi-agent conversation framework shows promise in enhancing diagnostic accuracy in diagnostically challenging medical scenarios.

## Introduction

Human cognitive biases in clinical decision-making are increasingly recognized as a crucial factor in health care errors and suboptimal patient outcomes [1]. These biases stem from innate psychological tendencies and can potentially lead to misjudgments and suboptimal outcomes in patient care [2-4]. Despite concerted efforts involving educational strategies, optimal work environments, and a culture promoting bias awareness and correction [5,6], the eradication of these biases remains an elusive goal.

The integration of artificial intelligence (AI), and in particular, large language models (LLMs), into clinical medicine is on the horizon [7]. LLMs have advanced text generation capability and extensive domain-specific knowledge [8,9]. Notably, these models have demonstrated their proficiency by successfully passing advanced medical examinations [10] and scoring clinical risk gradings on par with experienced physicians [11].

However, the deployment of LLMs in actual clinical diagnosis and decision-making processes has been mired in controversy, primarily due to the high stakes involved. The use of AI in medical settings is not just a technological issue; it intersects with complex ethical, legal, and medical considerations [11-16]. The accuracy of ChatGPT making the correct emergency medicine diagnosis is still limited to 77% to 83% [17]. Thus, concerns centering around the legal implications and accountability in cases where AI-driven diagnostics might lead to errors or misjudgments are a major hurdle.

This debate is rooted in the fundamental difference between human and machine intelligence. While LLMs can process and analyze vast quantities of data far beyond human capacity [18], they lack the nuanced understanding, empathy, and ethical reasoning inherent to human practitioners [19]. Human cognitive biases can be mitigated through a combination of awareness, education, and structured approaches [20]. Simulations of such discussions through LLM agents could provide a new solution to increase the accuracy of diagnosis [21]. The multi-agent framework features dialogue agents with near-human performance and could introduce an innovative paradigm in health care [22-24]. By simulating scenarios that mirror real-life clinical decision-making processes, and through reading the multi-agent conversations, clinicians can be made aware of potential cognitive biases and how to correct them. This facilitates learning in a controlled, educational environment [25,26].

Despite significant advancements in LLM technology, especially within multi-agent systems, its application to identify and mitigate human cognitive biases in clinical settings remains largely unexplored in current research. This study seeks to evaluate the efficacy of the multi-agent framework in achieving accurate final diagnoses following discussions on cognitive biases that may be present within the initial diagnosis. In addition, it aims to compare these outcomes with the differential diagnoses provided by experienced clinicians after reviewing the same scenarios. By doing so, the research aims to shed light on the potential of the multi-agent system to support clinical decision-making processes and enhance diagnostic accuracy in health care settings.

## Methods

**Overview**

We accessed GPT-4 [27] through an application programming interface call to the OpenAI server. The specific variant used was GPT-4 Turbo.

We implemented a comprehensive search strategy aimed at including all relevant reports on misdiagnoses attributed to cognitive biases. A selection of 15 case reports was identified after a full review of the published literature as a representative sampling.

### Ethical Considerations

Due to the nature of the study, institutional review board approval was not required, as the research did not involve patient data and did not constitute human participants.

### Search Strategy

In this study, we focused on case reports highlighting instances of misdiagnoses resulting from cognitive biases. Our research involved a comprehensive search of the PubMed database using the terms “case reports [Publication Type]” AND “cognitive bias[All Fields]”. PubMed was chosen for its comprehensive coverage of case studies across diverse medical disciplines, ensuring access to a broad spectrum of peer-reviewed case studies for our analysis.

Eligibility for inclusion requires case reports to meet four key criteria: (1) they must provide detailed case information sufficient for making the initial diagnosis; (2) they must include a final, accurate diagnosis for the patient; (3) the incorrect diagnosis must be linked to cognitive bias by the authors; and (4) the final diagnosis should not be a rare disease or unclear. A rare disease is a disease or condition that affects fewer than 200,000 patients per year. The list of exclusions for rare diseases was based on the National Organization for Rare Disorders [28]. We set no limits on the publication year of these reports.

Screening of abstracts for eligible studies was conducted by 2 independent clinically trained reviewers, YK and TXYL. Each reviewer separately assessed whether a case should be included or excluded based on predefined criteria. In instances of disagreement, SAL reviewed the justifications for exclusion and inclusion and made the final decision. The full texts were reviewed to obtain the case summary, the initial wrong diagnosis by the medical team, and the final diagnosis of the case reported.

For the studies included in the analysis, full-text extraction, including patient demographics, past medical history, initial presenting complaints, and results of the preliminary investigations, was conducted. For cases involving imaging data, such as chest x-rays, we did not incorporate the actual images into the query. Instead, we opted to include the legends or descriptions accompanying these images. In defining the boundaries of the clinical scenarios for our study, we restricted the information to that available up to the point of and before the initial diagnosis. This meant deliberately excluding any subsequent investigations, treatments, or management strategies that followed.

As GPT-4 Turbo has a knowledge base trained up to April 2023 [29], there is potential bias stemming from the inclusion of case reports that might have been part of the LLM’s pre-training data. Hence a personal clinical scenario that was not published on the internet was included. This complex case was derived from the critical care attending’s personal experience where cognitive biases had resulted in wrong and delayed diagnosis. A concise summary of these clinical scenarios, including the unique case, is provided in Multimedia Appendix 1.

### Multi-Agent Conversation Framework

In this study, we used the multi-agent conversation framework provided by AutoGen [22] to assess its efficacy in mitigating cognitive biases in clinical decision-making. Within the system, each agent interacts based on their predefined role prompts, thereby simulating the collaborative decision-making process typically observed among health care professionals.

The suggested optimal group size to facilitate group discussion and performance has been proposed to be between 3 and 5 [30]. In the absence of established literature recommending an optimal team size for mitigating cognitive biases in medical settings, we constructed a simulation using 3 to 4 different agents, representing a typical clinical team composition [31]. These configurations aim to realistically emulate the dynamics of clinical teams and their potential to reduce cognitive biases.

As shown in Table 1, we tested 3 different frameworks to identify the most effective configuration. Framework 3 consisted of 3 agents (Junior Resident I, Junior Resident II, and Recorder), while Frameworks 4 (Junior Resident I, Junior Resident II, Professional Expert, and Recorder) and 4-C (Junior Resident I, Junior Resident II, Senior Doctor, and Recorder) each utilized 4 agents, with the fourth agent playing different roles. The distinguishing feature of Framework 4-C, in contrast to other frameworks, lies in its explicit directive for the Senior Doctor role to engage in discussions specifically focused on cognitive biases alongside the initial diagnosis. In addition, we experimented with combinations involving 5 or more agents, but the fifth agent did not effectively participate in the conversations despite modifications to the prompts. Consequently, the frameworks were limited to a maximum of 4 agents. All prompts for agent roles can be found in Multimedia Appendix 2.

**Table 1:** Different roles in the multi-agent conversation framework. Framework 3 consists of 3 agents, and Frameworks 4 and 4-C consist of 4 agents each.

Agents present	Role descriptions	Multi-agent framework		
		3	4	4-C
Junior Resident I	To make the final diagnosis after considering the discussions	✓	✓	✓
Junior Resident II	The devil’s advocate and correct confirmation and anchoring bias	✓	✓	✓
Professional Expert	The field expert in any subspecialization required (eg, radiologists and cardiologists)		✓	
Senior Doctor	The tutor and facilitator of the discussion to reduce premature closure bias			✓
Recorder	To record and summarize the findings	✓	✓	✓

**Figure 1:**
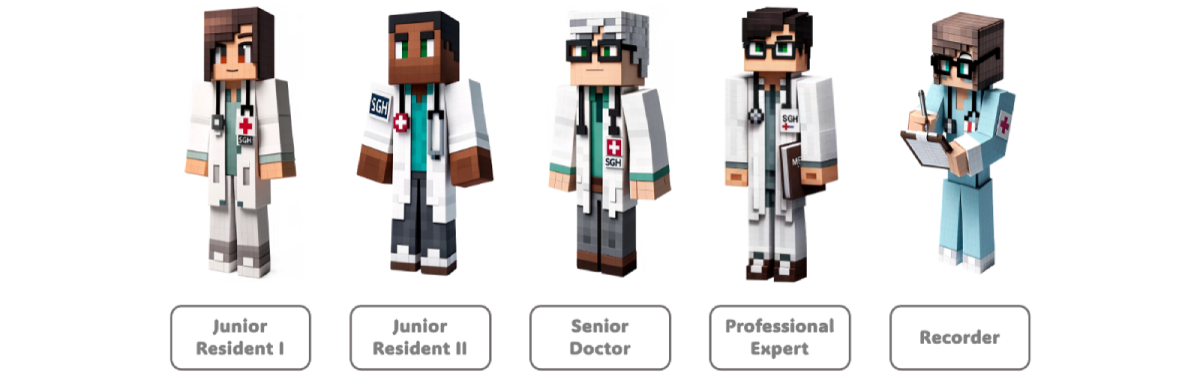
Different roles in the multi-agent conversation framework.

The diagnostic process was orchestrated through the collaborative efforts of simulated medical professionals (agents) with varying levels of expertise, as shown in Figure 1. Junior Resident I, as the primary physician, was tasked with presenting the initial diagnosis. Junior Resident I was given the personality of making swift assumptions but is willing to embrace feedback and consider alternative diagnostic possibilities. After the group discussion, Junior Resident I is then allowed to reconsider the most probable differential diagnosis along with an alternative. Junior Resident II, a colleague of Junior Resident I, critically appraised the initial diagnosis, pinpointing inconsistencies and advocating for alternative differential diagnoses. This role was instrumental in addressing potential confirmation and anchoring biases in the diagnostic process. Complementing the juniors, the Senior Doctor brought in-depth experience to the table, crucially identifying cognitive biases in the initial diagnosis and steering the junior residents toward a more nuanced and accurate diagnosis, while the Professional Expert aims to provide any specialist knowledge required to help with the diagnosis without further encouraging discussions of cognitive biases. This guidance was vital in circumventing premature diagnostic closure and knowledge bias. In addition, the role of the Recorder was to distill the outcomes of the discussion, compiling a definitive list of differential diagnoses and extracting key learning points from the collaborative effort, thereby enriching the diagnostic process with a comprehensive and multifaceted approach.

### Diagnostic Accuracy Assessment

The final accurate diagnoses for the clinical scenarios were extracted directly from the published cases. Summarized answers from both the multi-agent framework and human evaluators were marked as “Correct” if they matched the final accurate diagnoses. Vague answers, such as diagnosing “septic shock” when the accurate diagnosis was “endometriosis,” were marked as “Incorrect.” A total of 2 physicians graded the answers. In cases where there were discrepancies in their assessments, discussions were held to reach a consensus.

The overall performance of the framework was evaluated based on the accuracy of (1) the “initial diagnosis” made without any multi-agent discussions and (2) the “final diagnosis” after the discussions. Each clinical scenario within each multi-agent framework was simulated 5 times to assess the consistency of diagnoses across multiple iterations.

A total of 3 doctors, each with at least 5 years of clinical experience, were asked to provide their top differential diagnosis along with 2 other differentials based on the clinical scenarios. If the top differential was correct, both the “initial diagnosis” and “final diagnosis” were marked as correct. The diagnoses generated by the multi-agent framework were compared to those provided by human doctors using Fisher exact test.

### Bias Identification and Mitigation

**Figure 2:**
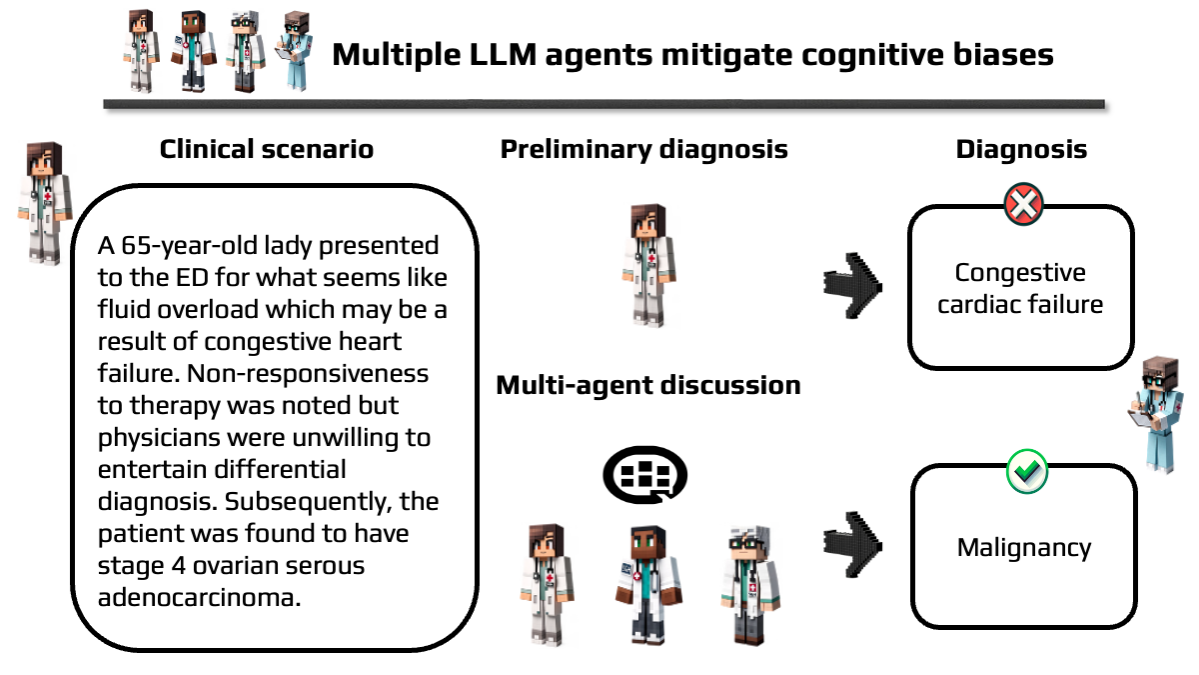
Schematic illustration of multi-agent (Framework 4-C) discussion dynamics leading to accurate differential diagnosis. ED: emergency department; LLM: large language model.

An integral part of the evaluation involved documenting the specific cognitive biases identified and addressed during the agents’ discussions. This aspect focused on understanding how effectively the multi-agent system could recognize and mitigate cognitive biases, which are crucial factors in diagnostic accuracy. Hallucinations are characterized by the dissemination of false medical information during multi-agent conversations or responses that fail to directly address the queries posed. This determination was made following an independent review by 2 doctors who thoroughly evaluated the provided answers. The interaction and decision-making process among the agents are illustrated in (Figure 2). This representation aids in visualizing the dynamics of the simulation and the interplay between different agents in reaching a diagnosis.

## Results

### Overview

**Figure 3:**
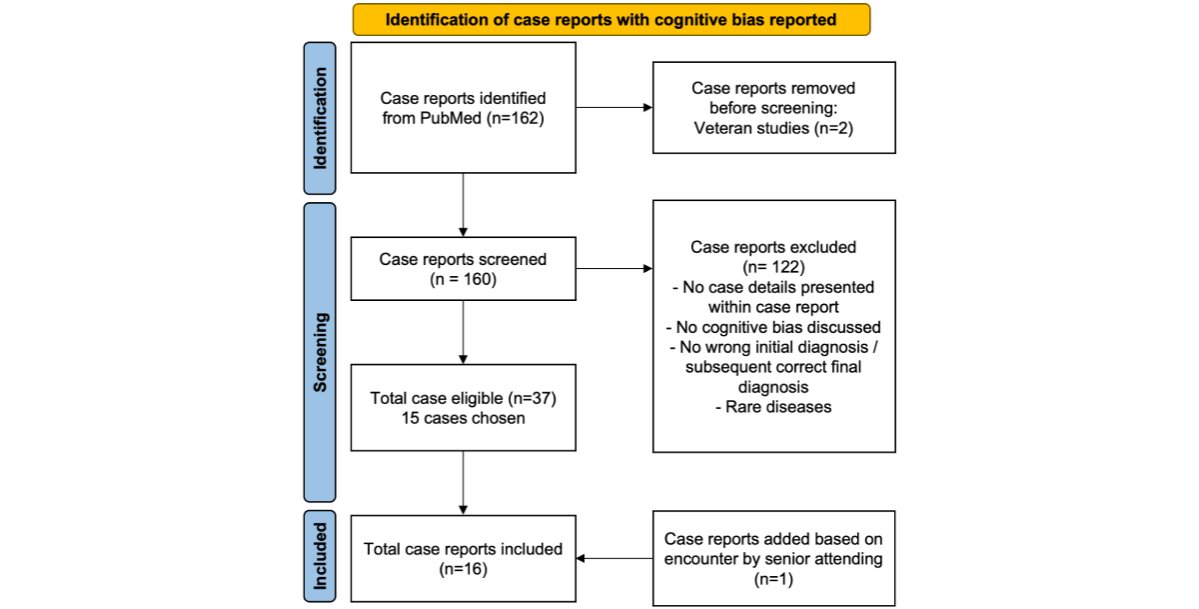
Flow diagram for identification of case reports with cognitive bias.

A comprehensive search of the PubMed database yielded 162 case reports, of which 37 were determined to be eligible for inclusion in the study. From this subset, 15 cases were selected for evaluation as a representative sampling. In addition, a 16th scenario, derived from critical care attending personal clinical experience, was included. The flow diagram can be viewed in Figure 3.

### Overall Performance of Multi-Agent Conversation Framework

A total of 240 responses were generated by the multi-agent conversation framework, encompassing both initial and final diagnoses following discussions with the agents. The initial diagnosis made by the first-responder agent had a correctness rate of 0% (0/80) across all 3 multi-agent frameworks, whereas the human-generated answers had an accuracy rate of 27% (13/48). After the multi-agent discussions, the final diagnosis was correct in 76% (61/80) of cases in Framework 4-C, which was significantly better than the human answers (odds ratio 3.49; *P*=.002), as shown in Table 2.

**Table 2:** Number of correct responses across different multi-agent frameworks and humans.

Multi-agent framework	Initial diagnosis, n (%)	Final diagnosis, n (%)	Odds ratio^a^	*P* value^a^
3 (n=80)	0 (0)	51 (64)	1.91	.10
4 (n=80)	0 (0)	55 (69)	2.39	.03
4-C (n=80)	0 (0)	61 (76)	3.49	.002
Human (n=48)	13 (27)	23 (48)	—^b^	—

^a^Fisher exact test.

^b^Not applicable.

### Clinical Scenarios

The clinical cases covered a broad spectrum of medical fields, ranging from pediatric to malignancy diagnosis. Specifically, 6 cases were centered on infectious disease diagnosis, 3 pertained to critical care, and 2 involved vascular-related diagnoses. The rest were diverse, spanning neurology, gynecology, cancer, urology, and endocrinology (Table 3). A notable aspect of these cases was the cross-disciplinary nature of most initial and final diagnoses, observed in 12 (75%) out of the 16 cases. For example, in one illustrative case (case 1 [32]), an older adult patient presented with symptoms of shortness of breath on exertion and cough, leading to an initial misdiagnosis of heart failure. However, further investigation, considering her ongoing treatment with infliximab for rheumatoid arthritis, revealed the actual diagnosis of miliary tuberculosis. Another case (case 12 [33]) involved a young woman presenting with sudden, left-sided sharp pleuritic chest pain, which lessened when sitting forward. Despite the initial chest radiograph being interpreted as showing no acute abnormalities, the AutoGen system, provided with this potentially misleading information, initially diagnosed the condition as a pulmonary embolism. Yet, after a thorough discussion and re-evaluation, the correct diagnosis of pneumothorax was established, indicating a missed finding in the chest radiograph.

**Table 3:** Clinical scenarios with the initial wrong and the final correct diagnosis are given in the scenarios.

Reference	Publication year	Initial wrong diagnosis	Final correct diagnosis
[32]	2015	Heart failure	Miliary tuberculosis
[34]	2017	Bronchial asthma triggered by bacterial pneumonia	Heart failure secondary to dilated cardiomyopathy
[35]	2018	Syndrome of inappropriate secretion of antidiuretic hormone	Nonfunctioning macropituitary adenoma causing adrenal insufficiency
[36]	2019	Headache and neck pain—migraines and muscle strain	Cryptococcal meningitis
[37]	2020	Complex regional pain syndrome flare	Left common and external iliac artery occlusion
[38]	2022	Pelvic inflammatory disease	Atypical ectopic pregnancy
[39]	2022	COVID-19 pneumoniae	Bacterial pneumonia (legionella pneumoniae)
[40]	2022	Urinary tract infection with complicated pyelonephritis	Vesicointestinal fistula due to Crohn disease
[41]	2022	Bone (sternum) tuberculosis	Syphilitic gumma and osteomyelitis
[42]	2022	Urinary tract infection	Vertebral osteomyelitis and bilateral psoas and retroperitoneal abscesses
[43]	2022	Anaphylaxis secondary to henna	Superior vena cava syndrome
[33]	2023	Pulmonary embolism	Pneumothorax
[44]	2023	Diabetic ketoacidosis with infections	Thiamine deficiency
[45]	2023	Endometritis	Ischemic bowel
[46]	2023	Acute myocarditis likely due to MIS-C^a^	Acute myocarditis caused by invasive bacterial infection
—^b^	—	Congestive cardiac failure	Malignancy

^a^MIS-C: multisystem inflammatory syndrome in children.

^b^Not applicable.

### Consistency of Multi-Agent Conversation Framework

There were variations observed in the repeated scenarios, particularly in the process of generating the top 2 differential diagnoses. For instance, in case 13 [44], a young patient presenting with lactic acidosis was initially diagnosed with diabetic ketoacidosis, and further discussions within the multi-agent environment led to the identification of thiamine deficiency. In 3 (60%) out of 5 simulations, Junior Resident I identified thiamine deficiency as the top differential diagnosis following the multi-agent discussions. In the remaining 2 simulations, gastrointestinal disorders were initially considered the most likely diagnosis, with thiamine deficiency being the second most likely differential.

The multi-agent discussions led to the correct final diagnosis in 13 (81%) out of the 16 scenarios across all 3 multi-agent frameworks. Furthermore, these discussions were effective in identifying various clinical biases, including anchoring bias, confirmation bias, availability bias, and premature closure. A detailed breakdown of the answers and the cognitive biases identified is provided in Multimedia Appendix 1. The detailed breakdown of correct answers for each scenario is available in Multimedia Appendix 3.

## Discussion

### Principal Findings

This study assessed the effectiveness of the multi-agent conversation framework in improving diagnostic accuracy and mitigating cognitive biases in clinical decision-making. Our findings reveal that integrating multi-agent discussions substantially enhances diagnostic accuracy. The best-performing framework, which used 4 agents, included 1 agent specifically tasked with identifying cognitive biases. This 4-C multi-agent framework that includes discussions on cognitive biases performed significantly better compared with human-generated answers. However, it is important to note that while 4 agents performed best in our study setting, this may vary in general applications.

The increase in diagnostic accuracy demonstrates the value of multi-perspective analysis in medical diagnosis, a core feature of the multi-agent conversation environment. This is in line with previous research emphasizing the importance of collaborative decision-making in health care to mitigate individual cognitive biases [47]. The consistency of responses in the repeated scenarios further validates its reliability and potential applicability in real-world clinical settings.

The effectiveness of the multi-agent conversation system, particularly in scenarios involving misleading or misinterpreted initial investigations, is noteworthy. This was exemplified in case 12, where our multi-agent framework successfully identified a pneumothorax that had been initially overlooked by human clinicians, and improved the accuracy of the final diagnosis to 100% (5/5) in multi-agent framework 4-C. The multi-agent systems were able to critically examine and question potential misinterpretations. Such capabilities are crucial in refining the diagnostic process and enhancing accuracy. While Brown et al [33] discussed the role of AI in the identification of pneumothorax, there is a potential for pre-trained LLMs to replace the decision aid, rather than to develop new resource-intensive systems such as image deep learning. This strategy aligns with the current trajectory of AI development in health care, where the focus is on integrating and maximizing existing AI technologies to enhance clinical decision-making and focus on sustainable AI [48,49].

The integration of multi-agent frameworks in clinical practice holds promising implications for enhancing diagnostic accuracy and ultimately improving patient outcomes. By systematically addressing cognitive biases through collaborative discussions among agents, these frameworks offer a structured approach to refining diagnostic reasoning. This approach not only complements traditional diagnostic methods but also introduces a dynamic element that challenges and verifies initial clinical hypotheses. In practical terms, the ability of multi-agent systems to consistently identify and correct potential diagnostic errors, as demonstrated in our study, suggests a transformative potential in reducing patient morbidity and optimizing treatment strategies. Furthermore, the application of these frameworks within electronic medical records (EMRs) could revolutionize decision-making processes by providing real-time, data-driven insights that augment clinician judgment and ensure adherence to best practices. As health care systems evolve toward more integrated and technology-driven approaches, the strategic incorporation of multi-agent systems stands poised to contribute significantly to improving the quality and efficiency of patient care delivery.

The results of our study extend beyond the educational benefits of multi-agents, highlighting their potential for broader clinical integration. The reflective process fostered by engaging with LLMs in diagnosing and revising cases not only cultivates an educational atmosphere conducive to developing critical thinking skills but also suggests practical applications in clinical settings [50]. One notable avenue is the integration of multi-agent into EMR systems. This could enhance decision-making processes by providing real-time, data-driven insights and augmenting the cognitive capabilities of medical professionals. Such integration would not only streamline the diagnostic process but also aid in the identification of potential cognitive biases, thereby enhancing the quality of patient care. Furthermore, the incorporation of multi-agents in EMRs could facilitate continuous learning and improvement, ensuring that medical practitioners remain updated with the latest medical knowledge and best practices, crucial for maintaining high standards in patient treatment and care.

### Limitations

The study, while providing valuable insights into the potential application of multi-agents in clinical diagnostics, is subject to several limitations. First, the reliance on published case reports limits the breadth of clinical scenarios, potentially affecting the generalizability of our findings to broader medical practice. Second, the exclusion of visual data, such as medical imaging, confines our model’s diagnostic capabilities to text-based information, omitting a critical component of clinical diagnosis. In addition, the inherent biases present in the LLMs, based on their pre-training data, could have influenced the diagnostic suggestions. Meanwhile, the technical limitations inherent in LLMs, including their understanding of complex medical terminologies and nuances [51], may not match the expertise of experienced clinicians, possibly limiting the scope of applicability.

Future studies could assess the effectiveness and adaptability of the multi-agent framework in evolving clinical scenarios. More importantly, while LLMs have demonstrated potential as a valuable clinical aid in correcting cognitive biases, the implementation of such technology in health care necessitates rigorous ethical and regulatory oversight [52] and should continue to augment rather than replace the human clinician’s expertise [14].

### Conclusions

In conclusion, our study highlights the potential of multi-agents in enhancing diagnostic accuracy in clinical decision-making. The findings support the integration of advanced generative AI technology in educational and clinical settings as a tool for augmenting human decision-making. Future research should focus on the application of these systems in real-time clinical environments and their impact on patient outcomes. Moreover, ethical and legal considerations regarding the use of AI in health care need continued exploration to ensure patient safety and professional accountability.

## Appendix

Multimedia Appendix 1 Summary of clinical scenarios and the initial and final correct diagnosis, as well as examples of cognitive biases present.

Multimedia Appendix 2 Prompts for different agent roles.

Multimedia Appendix 3 Detailed breakdown of the correct answers within each scenario stratified by each multi-agent framework and human.

## Abbreviations

AI: artificial intelligence
EMR: electronic medical record
LLM: large language model
