# Supplementary material for: Mitigating Cognitive Biases in Clinical Decision-Making Through Multi-Agent Conversations Using Large Language Models: Simulation Study
Source: J Med Internet Res. 2024 Nov 19;26:e59439. doi: 10.2196/59439 (PMC11615553; doi:10.2196/59439)
Supplement: Multimedia Appendix 1 [file jmir_v26i1e59439_app1.docx]

**Multimedia Appendix 1.** Summary of clinical scenarios and the initial and final correct diagnosis, as well as examples of cognitive biases present.

| **Scenario** | **Summary** | **Initial diagnosis by clinicians** | **Final correct diagnosis** | **Cognitive biases present** |
| --- | --- | --- | --- | --- |
| 1 | Elderly patient presented with shortness of breath on exertion and cough. She was initially misdiagnosed as having heart failure. Medical reconciliation later reveals that she has been taking infliximab for her RA. Subsequent investigations such as a CT thorax, sputum cultures from a bronchoscopy, and a normal echocardiogram reveal that she has miliary TB. | Heart failure | Miliary tuberculosis | Availability bias  Attribution bias  Premature closure |
| 2 | 23-month-old girl presenting with wheezing, fever, cough, and rhinorrhoea was initially misdiagnosed as having an asthma attack triggered by pneumonia. The patient's symptoms initially seemed to improve with salbutamol and IV steroid administration, however, the patient's symptoms recurred, and she was re-presented to ED multiple times. During the repeat ED visits, the patient was repeatedly diagnosed and treated for asthma due to anchoring bias. Subsequent investigations showed that the patient had heart failure due to dilated cardiomyopathy. | Bronchial asthma triggered by bacterial pneumonia | Heart failure secondary to dilated cardiomyopathy | Anchoring bias  Diagnosis momentum  Confirmation bias |
| 3 | A 50-year-old male with hyponatremia was wrongly diagnosed with SIADH due to anchoring bias and urine studies that supported their preliminary diagnosis. SIADH should be a diagnosis of exclusion after ruling out other causes such as adrenal and thyroid aetiologies, including hypothyroidism and hypocortisoism. The patient was eventually diagnosed with a pituitary adenoma. | SIADH | Pituitary adenoma | Anchoring bias  Premature closure |
| 4 | A 33-year-old male with cryptococcal meningitis was misdiagnosed with migraines and muscle strain due to delayed HIV diagnosis failing to recognize immunosuppression. Even with the HIV diagnosis, the team attributed his persistent symptoms to muscle strain and migraine due to anchoring bias as he previously responded to symptomatic treatment. | Headache and neck pain - migraines and muscle strain  Nausea - gastritis | Cryptococcal meningitis | Availability heuristic Representativeness heuristic Diagnostic overshadowing |
| 5 | 45-year-old female with a background of Behcet's disease and complex regional pain syndrome presents with a complex left lower limb hyperalgesia, oedema, and skin temperature changes. She was wrongly diagnosed due to anchoring bias by her physicians as her symptoms seem to fit her already established diagnosis of complex regional pain syndrome. Her team was late to consider an arterial thrombosis although it also fit the patient's symptoms as it is rare. | Complex regional pain syndrome flare | Left common and external iliac artery occlusion | Anchoring bias  Premature closure  Overconfidence bias  Diagnosis momentum |
| 6 | a 25-year-old patient who had an atypical ectopic pregnancy but was misdiagnosed as a pelvic inflammatory disease due to resolution of pain and tachycardia with treatment. | Pelvic inflammatory disease | Atypical ectopic pregnancy | Anchoring bias  Premature closure |
| 7 | A patient who presented with pneumonia symptoms during the COVID-19 pandemic. He was initially suspected of having COVID.  Due to persistent hyponatremia and negative COVID swabs, other differential diagnosis was considered later and tested positive for legionella subsequently. | COVID-19 pneumoniae. | Bacterial pneumonia (Legionella pneumoniae) | Availability bias  Representativeness bias Premature closure |
| 8 | 21-year-old male was referred from another hospital for what seemed like a poorly responding complicated UTI despite prolonged antibiotic treatment. The physicians failed to investigate the cause of the complicated UTI which turned out to be a vesicointestinal fistula due to Crohn's disease. | Urinary tract infection (UTI) with complicated pyelonephritis | Vesicointestinal fistula due to Crohn's disease | Anchoring bias  Premature closure |
| 9 | A 30-year-old bisexual male, with a significant history of recurrent syphilis infections, presenting with chest pain and MRI findings of erosive lesions on the sternum and left ninth rib was wrongly treated for bone TB as the physicians failed to consider syphilis osteomyelitis as the patient did not present with any signs of early syphilis. This atypical presentation of syphylis osteomyelitis is typical in HIV patients. | Bone (sternum) TB | Syphilitic gumma and osteomyelitis | Availability heuristic  Diagnostic momentum  Representativeness bias |
| 10 | 83-year-old male presenting with acute on chronic lower back pain and fever was wrongly diagnosed with UTI due to anchoring bias with a pyuria urine sample despite him having no UTI symptoms and multiple lower back pain "red flags". Patient was eventually diagnosed with vertebral osteomyelitis. | Urinary tract infection | Vertebral osteomyelitis and bilateral psoas and retroperitoneal abscesses | Premature closure  Representativeness heuristic |
| 11 | 53-year-old female presenting with a 3-day history of difficulty breathing, face and neck swelling misdiagnosed with anaphylaxis. The physicians failed to consider that her symptoms with a history of rectal cancer and vascular port-a-cath situ, SVC obstruction was a likely diagnosis. | Anaphylaxis secondary to Henna | Superior vena cava syndrome | Anchoring bias  Availability heuristic  Confirmation bias |
| 12 | A 27-year-old presented with chest pain and a positive D dimer test. Was initially wrongly diagnosed with pulmonary embolism and started treatment. Subsequently, further testing showed that she actually had a pneumothorax, which the findings were originally missed in the chest XR. | Pulmonary embolism | Pneumothorax | Confirmation bias  Availability bias |
| 13 | A young woman was admitted for diabetic ketoacidosis with persistent, asymptomatic lactic acid elevation during the evolving COVID-19 pandemic. Cognitive biases in interpreting an elevated LA in this patient’s care resulted in an extensive infectious workup instead of the low-cost and potentially diagnostic provision of empiric thiamine | DKA with infection | Thiamine deficiency | Anchoring bias  Availability bias  Premature closure |
| 14 | A 29-year-old lady who had a recent abortion and came in with abdominal pain. The initial diagnosis of endometritis for her subsequently was actually abdominal compartment syndrome and ischemic bowel. | Endometritis | Ischemic bowel | Availability heuristic  Confirmation bias  Diagnosis momentum  Representativeness heuristic |
| 15 | 6-year-old boy with myocarditis caused by invasive meningococcal infection, which was initially misdiagnosed as MIS-C due to diagnostic bias amid the COVID-19 pandemic as the patient fulfilled 5 of 7 of the CDC and 7 of 10 of the WHO criteria for MIS-C. | Acute myocarditis likely due to MIS-C | Acute myocarditis caused by invasive bacterial infection | Anchoring bias  Premature closure |
| 16 | A 65-year-old lady presented to the ED for what seems like fluid overload which may be a result of congestive heart failure. Non-responsiveness to therapy was noted but physicians were unwilling to entertain differential diagnosis. Subsequently, the patient was found to have stage 4 ovarian serous adenocarcinoma. | Congestive cardiac failure | Malignancy | Availability bias  Confirmation bias  Framing effect |

RA; Rheumatoid Arthritis, TB; Tuberculosis, ED; Emergency Department, SIADH; Syndrome of inappropriate secretion of diuretic hormone, UTI; Urinary Tract Infection, HIV; Human Immunodeficiency Virus, SVC; Superior Vena Cava, MIS-C; Multisystem inflammatory syndrome in children, CDC; Centers for Disease Control and Prevention, WHO; World Health Organization.
