# Supplementary material for: Mitigating Cognitive Biases in Clinical Decision-Making Through Multi-Agent Conversations Using Large Language Models: Simulation Study
Source: J Med Internet Res. 2024 Nov 19;26:e59439. doi: 10.2196/59439 (PMC11615553; doi:10.2196/59439)
Supplement: Multimedia Appendix 3 [file jmir_v26i1e59439_app3.docx]

**Multimedia Appendix 3.** Detailed breakdown of the correct answers within each scenario stratified by each multi-agent framework and human.

| Multi-agent framework | 3 | | 4 | | 4-C | | Human | |
| --- | --- | --- | --- | --- | --- | --- | --- | --- |
| Scenario | Initial | Final | Initial | Final | Initial | Final | Initial | Final |
| 1 | 0.0 | 0.0 | 0.0 | 0.0 | 0.0 | 0.0 | 0.0 | 0.0 |
| 2 | 0.0 | 0.0 | 0.0 | 0.0 | 0.0 | 0.0 | 33.3 | 66.7 |
| 3 | 0.0 | 20.0 | 0.0 | 60.0 | 0.0 | 60.0 | 0.0 | 0.0 |
| 4 | 0.0 | 100.0 | 0.0 | 100.0 | 0.0 | 100.0 | 66.7 | 100.0 |
| 5 | 0.0 | 100.0 | 0.0 | 80.0 | 0.0 | 100.0 | 33.3 | 66.7 |
| 6 | 0.0 | 100.0 | 0.0 | 100.0 | 0.0 | 100.0 | 33.3 | 66.7 |
| 7 | 0.0 | 100.0 | 0.0 | 100.0 | 0.0 | 100.0 | 66.7 | 100.0 |
| 8 | 0.0 | 100.0 | 0.0 | 100.0 | 0.0 | 100.0 | 66.7 | 100.0 |
| 9 | 0.0 | 40.0 | 0.0 | 60.0 | 0.0 | 100.0 | 0.0 | 33.3 |
| 10 | 0.0 | 100.0 | 0.0 | 100.0 | 0.0 | 100.0 | 33.3 | 33.3 |
| 11 | 0.0 | 100.0 | 0.0 | 100.0 | 0.0 | 100.0 | 66.7 | 66.7 |
| 12 | 0.0 | 60.0 | 0.0 | 40.0 | 0.0 | 100.0 | 0.0 | 66.7 |
| 13 | 0.0 | 100.0 | 0.0 | 100.0 | 0.0 | 100.0 | 0.0 | 0.0 |
| 14 | 0.0 | 20.0 | 0.0 | 0.0 | 0.0 | 0.0 | 0.0 | 0.0 |
| 15 | 0.0 | 0.0 | 0.0 | 60.0 | 0.0 | 60.0 | 0.0 | 33.3 |
| 16 | 0.0 | 80.0 | 0.0 | 100.0 | 0.0 | 100.0 | 33.3 | 33.3 |
| Average Correct | 0.0 | 63.8 | 0.0 | 68.8 | 0.0 | 76.3 | 27.1 | 47.9 |
| Hallucinations | 0.0 | | 0.0 | | 0.0 | | - | - |
